# Dilong: Role in Peripheral Nerve Regeneration

**DOI:** 10.1093/ecam/neq079

**Published:** 2011-06-15

**Authors:** Yung-Ming Chang, Wei-Yi Chi, Tung-Yuan Lai, Yueh-Sheng Chen, Fuu-Jen Tsai, Chang-Hai Tsai, Wei-Wen Kuo, Yi-Chang Cheng, Chien-Chung Lin, Chih-Yang Huang

**Affiliations:** ^1^School of Chinese Medicine, China Medical University, Taichung, Taiwan; ^2^The Pre-Clinical Medicine College, Nanjing University of Traditional Chinese Medicine, Nanjing 210029 Jiangsu, China; ^3^Drexel University, School of Nursing and Health Professions, Department of Physical Therapy and Rehabilitation Sciences, Philadelphia, PA, USA; ^4^School of Post-Baccalaureate Chinese Medicine, China Medical University, Taichung 404, Taiwan; ^5^Graduate Institute of Chinese Medical Science, China Medical University, Taichung 404, Taiwan; ^6^Department of Healthcare Administration, Asia University, Taichung, Taiwan; ^7^Department of Biological Science and Technology, China Medical University, Taichung, Taiwan; ^8^Emergency Department, Taichung Veterans General Hospital, Taiwan; ^9^Orthopaedic Department, Armed Forces General Hospital, Taichung, Taiwan; ^10^Graduate Institute of Basic Medical Science, China Medical University, Taichung 404, Taiwan; ^11^Department of Health and Nutrition Biotechnology, Asia University, Taichung 413, Taiwan

## Abstract

Dilong, also known as earthworm, has been widely used in traditional Chinese medicine (TCM) for thousands of years. Schwann cell migration and proliferation are critical for the regeneration of injured nerves and Schwann cells provide an essentially supportive role for neuron regeneration. However, the molecular mechanisms of migration and proliferation induced by dilongs in Schwann cells remain unclear. Here, we discuss the molecular mechanisms that includes (i) migration signaling, MAPKs (mitogen-activated protein kinases), mediated PAs and MMP2/9 pathway; (ii) survival and proliferative signaling, IGF-I (insulin-like growth factor-I)-mediated PI3K/Akt pathways and (iii) cell cycle regulation. Dilong stimulate RSC96 cell proliferation and migration. It can induce phosphorylation of ERK1/2 and p38, but not JNK, and activate the downstream signaling expression of PAs (plasminogen activators) and MMPs (matrix metalloproteinases) in a time-dependent manner. In addition, Dilong stimulated ERK1/2 and p38 phosphorylation was attenuated by pretreatment with chemical inhibitors (U0126 and SB203580), and small interfering ERK1/2 and p38 RNA, resulting in migration and uPA-related signal pathway inhibition. Dilong also induces the phosphorylation of IGF-I-mediated PI3K/Akt pathway, activates protein expression of PCNA (proliferating cell nuclear antigen) and cell cycle regulatory proteins (cyclin D1, cyclin E and cyclin A) in a time-dependent manner. In addition, it accelerates G_1_-phase progression with earlier S-phase entry and significant numbers of cells entered the S-phase. The siRNA-mediated knockdown of PI3K that significantly reduces PI3K protein expression levels, resulting in Bcl_2_ survival factor reduction, revealing a marked blockage of G_1_ to S transition in proliferating cells. These results reveal the unknown RSC96 cell migration and proliferation mechanism induced by dilong, which find use as a new medicine for nerve regeneration.

## 1. Introduction

### 1.1. Regeneration of Nerves

Nerve regeneration is a complex phenomenon that has interested scientists for many years. Neurons can be separated into central and peripheral nervous systems (PNSs), which have different anatomical structures and regenerative ability. In mammals, the central neurons without a myelin sheath are difficult to regenerate. In contrast to the central nervous system, the PNS with a myelin sheath exhibit easier regrowth [1]. Regrowth ability results from intrinsic neuronal activities and surrounding non-neuronal properties in which Schwann cells provide an essentially supportive activity for neuron regeneration. Schwann cells are the supporting cells of the PNS and can differentiate into the myelin sheath of the PNS and proliferate and migrate into the distal end of the injured nerve area [2]. Moreover, Schwann cell migration, which also occurs at the proximal end of the injured area, provides a guide for regenerating axons by interacting with nerve fibers or basal lamina [3]. Since Schwann cell migration is critical for axonal elongation and remyelination of injured nerves [3, 4], those factors that regulate Schwann cell migration have been widely investigated. Peripheral nerve injury locally activates Schwann cells and macrophages to synthesize a cocktail of neurotrophic factors, adhesion molecules, cytokines and growth-promoting surface molecules [5, 6]. However, the mechanisms of action of these regulating factors on Schwann cell migration, proliferation and signal transformation remain unclear.

### 1.2. Pathways that Play a Role in Cellular Proliferation and Migration

The mitogen-activated protein kinase (MAPK) family plays an essential role in inducing cell proliferation [7] and migration [8]. Extracellular signal-regulated protein kinase (ERK) that belongs to MAPK family has been studied extensively [9]. Results reveal that ERK is related to migration of various cell types, including fibroblasts and carcinoma cells [10, 11], but not in Schwann cells. Recently, several studies found that after nerve injury, the increased activation of ERK [12] phosphorylation promotes neurite outgrowth [13]. Interestingly, to promote migration, growth cones at the tip of an axon secrete proteases that are thought to dissolve cell-cell and cell-matrix adhesions during peripheral nerve regeneration. These proteases include the plasminogen activators (PAs), tissue PA (tPA) and urokinase PA (uPA) and their substrate, plasminogen [14]. Many experiments have determined that after injury, a rapid increase of tissue PA expression has been observed in neurons [14, 15]. Tissue PA or uro kinase PA activates plasmin that consequently activates MMP-9 and MMP-2 [16]. It has been shown that the lack of plasminogen activators affects MMP-9 and MMP-2 activity [17]. However, little is known about Schwann cell migration using MEK/ERK signaling pathways to active PAs and MMPs. In addition, accumulating evidence has also indicated that c-Jun NH2-terminal kinase (JNK) and p38, the other two members of the MAPK super family, have the affection on cell migration regulation [10]. To promote migration in cells, the expression of matrix-degrading proteolytic enzymes (PAs and MMPs) could be regulated by JNK [18] and p38 [19] signal transduction pathways.

## 2. Role of Growth Factors

### 2.1. Insulin-Like Growth Factors

Insulin-like growth factor-I (IGF-I) is a polypeptide hormone synthesized by proliferating Schwann cells [20]. The secretion of IGF-I is controlled by the growth hormone [21]. In response, IGF-I stimulates the growth and differentiation of fetal neurons [22] and increases neurite sprouting and outgrowth *in vitro* [23, 24]. Interestingly, IGF-I not only stimulates proliferation but also promotes survival in several cell types. It can rescue Schwann cells from apoptosis via PI3-K signaling, which is upstream from caspase activation [25]. *In vivo*, the signal cascade for early upregulation of IGF-I has been shown to promote retinal ganglion cells (RGCs) survival and axonal regeneration through the PI3K/Akt system after optic nerve injury in goldfish [26].

### 2.2. PI3K/Akt System and Peripheral Nerve Regulation

Many studies have investigated the effects of the PI3K/Akt system on peripheral nerve regeneration. IGF-I functions as a progression factor in the cell cycle [27], promoting G_1_/S cell cycle progression via the phosphatidylinositol 3-kinase/serine-threonine kinase (PI3K/Akt) pathway, resulting in DNA synthesis and cell proliferation [28]. This hormone protects neurons in the PNS from apoptosis by activating the PI3K/Akt pathway, which in turn phosphoralates Bad and activates Bcl_2_, an anti-apoptotic protein that interferes with the activation of caspases [26, 29, 30]. Furthermore, it has been suggested that the inhibition of PI3K activation can completely block Schwann cell proliferation and survival [31]. Generally, these data strongly indicate that IGF-I is an important molecule for controlling regeneration after nerve injury. Therefore, IGF-I has been used as a therapeutic target for the treatment of peripheral nerve injury and motor neuron diseases [32]. Insulin-like growth factor I (IGF-I) is currently in clinical trials for treatment of amyotrophic lateral sclerosis (ALS) based on its neuroprotective effect on motor neurons [33].

## 3. Traditional Chinese Medicine

### 3.1. Is There a Role for Dilong?

The pharmacology and clinical application of traditional Chinese medicine has been well documented for several thousand years. Recently, biomedical material science combined with Chinese herbal medicine has been applied to analyses of nerve regeneration. Several Chinese medicines have been identified as enhancing neuron regeneration. In Tsai's study, ginsenoside Rb1 (GRb1) filled into a silicon rubber chamber can bridge a 15-mm gap in injured rat sciatic nerves [34]. Therefore, the herbal medicine has a good potential for treating injured nerves. The earthworms, also called “dilong” in Chinese, are a widely used Chinese herbal medicine [35]. Extracting medicinal compounds from dilong has traditionally been practiced by indigenous people throughout the world, more particularly in Asia [36]. The potential treatment effects of earthworms may come from their dense soil-based nutritional content [37].

Bu Yang Huan Wu Tang (BYHWT), a Chinese herb complex prescription, has been applied for treating the sequelae of stroke, eye and mouth distortion, stiffness in the tongue and aphasia, as well as atrophy and paralysis of lower limbs. BYHWT consists of Dilong (Earthworm), Chi Shao (Peony Red), Tao Ren (Persica), Hong Hua (Carthamus), Dang Gui (Chinese Angelica Root), Huang Qi (Astragalus) and Chuan Xiong (Cnidium). It has been shown that BYHWT can increase the level of NO in cerebral infarct rats and reduce the area of cerebral infarct [38]. Furthermore, several research results indicate that BYHWT may promote the repair and regeneration of neurons and injured nerve fibers [39, 40]. After administered BYHWT for 4 weeks, the conduction velocity of pulses in the newly regenerative nerve of the treatment group was significantly faster than that of the control group. The blood vessel area around the regenerative nerves in the treatment group was also considerably larger. These results provide many information for further studies on the role of Dilong in nerve regeneration.

### 3.2. Evidence for Diverse Role of Dilong

Previous studies of dilong have shown its antimicrobial [41], hepatoprotective [42], anticancer and scar wound-healing characteristics [35]. The anti-inflammatory activity together with anti-oxidant properties may be due to the high polyphenolic content in dilong tissue [42]. Moreover, crude dilong extract has a thrombolytic effect that could significantly promote blood circulation and remove stasis [43]. In healthy human volunteers, orally administered dilong powder increased levels of tissue plasminogen activator and fibrinolytic activity [44]. These results suggest that earthworm powder represents a possible oral thrombolytic agent.

### 3.3. Hypothetical Role of Dilong in Nerve Regulation

*In vivo* experiments have also found that a mixed prescription of liquid extracted from dilong more obviously improves peripheral nerve regeneration than icariin [45]. Therefore, many scientists focus on purifying the contents of crude dilong powder. Different contents of dilong extract will be discussed as follows. First, Lumbrokinase extracted from dilong has been used to treat stroke and cardiovascular diseases [41]. Lumbrokinase is a group of proteolytic enzymes [36]. It includes a plasminogen activator and plasmin [46] that serve to activate plasminogen and dissolve fibrin directly [47]. Second, dilong tissue homogenates have revealed a glycolipoprotein mixture composed of macromolecules referred to as G-90. G-90 possesses several growth factors and also participates in tissue regeneration and wound healing [48]. Further experimental works are needed to more fully characterize the molecules and potential mechanisms of dilong extract involved in peripheral nerve regeneration.

**Figure 1:**
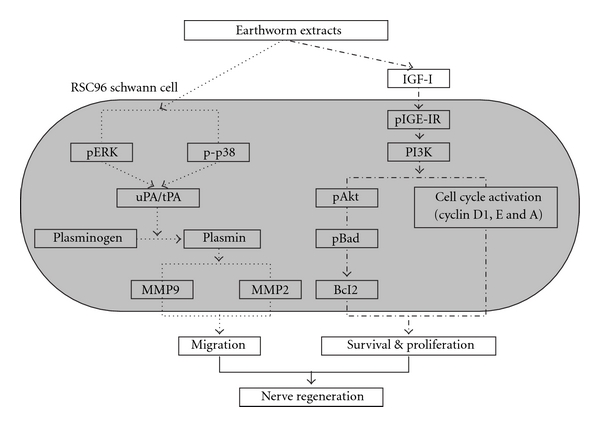
Schematic model of migrative survival and proliferative effects of Dilong extract on Schwann cell.

There is still no conclusive explanation for the possible molecular mechanism involved in Schwann cell migrating and proliferating events. Peripheral nerve regeneration requires a permissive environment and activation of the intrinsic growth capacity of neurons. Axon regrowth and remyelination of the regenerated axons by Schwann cells are both essential. Multiple factors including neurotrophic factors, extracellular matrix (ECM) proteins and hormones participate in Schwann cell dedifferentiation, proliferation and remyelination. A schematic model (Figure 1) is proposed to explain the migrative, survival and proliferative effects of dilong extract on Schwann cells. In this review article, we will discuss the peripheral nerve regeneration mechanisms, with particular attention to the proliferation and migration of Schwann cells induced by dilong.

## 4. Migration Signaling: MAPKs Mediated PAs and MMP2/9 Pathway

### 4.1. MAPK and Schwann Cell Migration

The MAPK family is a crucial regulator of pathways involved in cell proliferation [7] and migration [8]. JNK, p38 and ERK1/2, the members of MAPKs family, play crucial roles in nerve cells migration [10]. The whole MAPKs pathway is illustrated by the dotted line in Figure 1. This study further demonstrated that dilong extract stimulated ERK1/2 and p38, but not JNK activation in a time-dependent manner, leading to Schwann cell migration. Dilong-induced Schwann cell motility and phosphorylation of ERK1/2 and p38 were both attenuated by pretreatment with MEK1/2 (U0126) and p38 (SB203580) inhibitors. Transfection with siRNA of MEK1/2 and p38 significantly reduced migration in response to dilong extract in Schwann cells as well. The signaling migration pathway in earthworm-stimulated Schwann cells, inducing the activation of uPA and tPA mediated through the ERK1/2 and p38. To promote migration, cells secrete proteases (PA and uPA) that are thought to degrade matrix molecules and cell adhesion. The ERK1/2 and p38 phosphorylation leads to the expression of uPA and tPA that occurs in a time-dependent manner, during the elevation of MMP9 and MMP2 levels and activity. In addition, the highly expressed uPA in the epidermis of damaged tissue is also regulated by the fibroblast growth factor (FGF-2) that affects MAPK kinase (MEKK-1) and MEKK-1s downstream extracellular signal-regulated kinases (ERK1/2) for controlling uPA expression [49]. Another regulating factor is p38. In endothelial cell migration, the p38 MAPK pathway also participates in by regulating uPA expression [50].

### 4.2. Dilong Extract and Signaling

In Chang's study, results reveal that dilong extract enhances uPA expression directly through the ERK1/2 and p38 signaling pathway [51]. To promote migration, cells secrete proteases that are thought to degrade matrix molecules and cell adhesion. These proteases include tPA and uPA [14]. In contrast to PAs, PAI-1 is thought to be the main inhibitor of the plasminogen activators. Chang's study clearly shows that the phosphorylation of ERK1/2 and p38 accompanies the increased expression of uPA, but PAI-1 expression is gradually decreased. Interestingly, tPA levels reached the maximal early at 2 h, and then began to decline slightly until 20 h. It suggests that the maximum expression of tPA occurred early following 4 h treatment, because tPA is the main PAs in the nerve to facilitate growth cone movement by digesting extracellular matrices and cell adhesions. Pittman and DiBenedetto [52] reported that the over expressing tPA can regenerate neurites to a greater extent and migrate faster than the control group in complex extracellular matrix. Ulfhammer et al. [53] found that tPA activation could be mediated through p38 pathways, leading to an increase in tPA expression. Chang's experiments further show that SB203580 inhibited p38 phosphorylation and suppressed tPA protein expression in Schwann cells. Thus, tPA activation occurs not only through ERK1/2 activation but also through the p38 signaling pathway.

## 5. Proliferative and Survival Signaling: IGF-I-Mediated PI3K/Akt Pathways

The PI3K/Akt signaling mediated by IGF-I (dot-dashed line in Figure 1) plays an important role in cell proliferation as well as cell survival [14, 28, 30, 54]. IGF-I is a polypeptide hormone synthesized by proliferating Schwann cells [20] and it directly induces neurite outgrowth via a PI3K/Akt-dependent mechanism [26, 55]. Moreover, IGF-I rescues Schwann cells from apoptosis via PI3K signaling that is upstream from caspase activation [56], and also requires that PI3K/Akt-mediated progression from G1 to S phase of the cell cycle [54]. Conversely, PI3K inhibitors blocked the anti-apoptotic and protective effects of IGF-I, demonstrating that PI3K is essential for trophic factor-induced survival of Schwann cells [30, 56]. The plasma membrane is subsequently phosphorylated by PI3K. This activates the protein kinase Akt and the following steps of cell proliferation. IGF-1 seems to play an important role in controlling brain growth and cell number [57, 58]. IGF-1 injection in embryos increased rat cerebral cortex DNA content by 28%, suggesting a role for IGF-1 in control of brain growth. IGF-1 therefore seems to be a mitogen for cortical precursors during embryonic development [28].

## 6. Cell-Cycle Regulation

### 6.1. IGF-1

IGF-I has also been shown to function as a progression factor in the cell cycle by regulating expression levels [59]. It can promote G_1_/S cell cycle progression via the phosphatidylinositol 3-kinase/serine-threonine kinase (PI3K/Akt) pathway, which then results in DNA synthesis and cell proliferation. Chang's analysis indicates that treatment with dilong extract induces phosphorylation of the IGF-I-mediated phosphatidylinositol 3-kinase/serine-threonine kinase (PI3K/Akt) pathway, it activates protein expression of cell nuclear antigen (PCNA) in a time dependent manner [60]. Cell cycle progression is tightly regulated by a complex network of cell cycle regulatory molecules, such as cyclins. Proliferating cells pass through several cell cycle checkpoints, such as the G_1_ to S transitions. G1 checkpoint is considered to be the most important one in the replication of DNA and mitosis. The results show that G_1_ transits into the S phase in 12–16 h, and S transits into the G_2_ phase 20 h after exposure to dilong extract. Strong expression of cyclin D1, cyclin E and cyclin A occurs in a time-dependent manner. Progression through the first gap phase (G1) requires cyclin D and cyclin E activity [61]. Expression of these two cyclin proteins orchestrates the progression of cells through G_1_ and into the S-phase of the cell cycle [62, 63]. Cyclin A is required for DNA replication in the S-phase and in mitosis initiation (M-phase) [63].

### 6.2. Small Interfering RNA

Small interfering RNA (siRNA)-mediated knockdown of PI3K significantly reduced P13K protein expression levels, resulting in Bcl_2_ reduction of survival factor and a marked blockage of G_1_ to S transition in proliferating cells. These results demonstrate that dilong extract promotes the proliferation and survival of RSC96 cells via IGF-I signaling. The mechanism is primarily dependent on the PI3K protein [51]. Dilong extract promoted DNA replication and growth of RSC96 cells by upregulating the sequential expression of cyclin D1, cyclin E and cyclin A, thereby elevating the number of cells in the S phase in a time-dependent manner. Studies on the proliferative action of IGF-I in cultured fibroblasts (BALB/c-3T3) [64, 65] and mammary epithelial cells [66] indicate that IGF-I acts to stimulate progression through G_1_ or the G_0_/G_1_ transition. Chang's data are in agreement with the results of those studies revealing that the cell cycle is not only regulated by cyclins but also mediated by IGF-I [51]. Therefore, cell cycle alterations may be critical determinants of the increased proliferation potency-induced dilong extract.

## 7. Perspectives on Schwann Cell Migration

The mechanism in which dilong extract regulates Schwann cell migration, proliferation and survival is investigated in this article. Specific signaling pathways in dilong-stimulated Schwann cells are shown in Figure 1. For thousands of years dilong has been used as a drug for various diseases in China and the Far East [44]. Until now, the function of dilong extract on nerve regeneration is still unclear. The development and regeneration of the PNS is highly dependent on the migration of Schwann cells and the extension of axons toward their distant targets. Recently studies revealed that MAPKs, including JNK, p38 and ERK1/2, play crucial roles in nerve cell migration [10]. Dilong-induced Schwann cell motility and phosphorylation of ERK1/2 and p38 were both attenuated by pretreatment with MEK1/2 (U0126) and p38 (SB203580) inhibitors. In addition, transfection with siRNA of MEK1/2 and p38 significantly reduces migration in response to dilong extract in Schwann cells. Using inhibitors and siRNA, the migrative effects of dilong extract on Schwann cells were further identified to be ERK1/2 and p38 signaling dependent. These assays help researchers to examine the individual steps in the complex signaling cascades and clearly illustrate effects of direct dilong extract on Schwann cell migration. Dilong extract enhances uPA expression directly through the ERK1/2 and p38 signaling pathway. When cells treated with dilong extract resulted in ERK1/2 and p38 phosphorylation, the expression of uPA and tPA lead to elevated MMP9 and MMP2 levels and activity. Several experiments have indicated that dilong fibrinolytic enzyme acts as a plasminogen activator [67], suggesting a tPA-like function [68]. Another family of proteases, the matrix metalloproteases (MMPs), are also implicated in peripheral nerve regeneration [69], and involved in many cell migration phenomena and produced by many cell types, including neurons [70]. MMPs are secreted as inactive molecules and require activation via other proteases [71]. Plasmin, activated by tPA or uPA, can activate MMP-9 and MMP-2 [16]. A novel fibrinolytic enzyme isolated from the earthworm *Pheretima aspergillum* can directly and strongly dissolve human thrombi and fibrin, and also activate human plasminogen to plasmin.

## 8. Role of the Glycolipoprotein G-90

G-90, a biologically active glycolipoprotein complex, isolated from whole dilong tissue extract (*Eisenia foetida*) [61]. There are several function attributed to G-90, including an insulin-like growth factor (IGF like), an immunoglobulin-like growth factor (IgFG-like) and epidermal growth factor (EGF) [48] and mitogenicity [72]. G-90 also has an apparent protective effect against the toxicity of H_2_O_2_ and stimulated the growth of the cell [73] and show anticoagulative and fibrinolytic activities [74, 75]. The results revealed that G-90 also participates in tissue regeneration and wound healing. The glycolipoprotein tissue homogenate extract from the earthworms *Eiseni*a *foetida* (G-90) can activate signal transduction pathways, leading to wound healing. G-90 promote and cause an increased concentration of EGF and FGF as observed 6 h after wounding on mouse skin [76].

Stimulation of cell proliferation during nerve regeneration usually involves initiation and progressive activity of growth factors. As mentioned previously, these growth factors (GDNF, BDNF, FGF and NGF) can activate MAPK pathway to stimulate Schwann cell migration. Highly expressed uPA in the epidermis of damaged tissue is regulated by the FGF-2 that activates MAPK kinase (MEKK-1) and its downstream ERK1/2 [49]. Despite these encouraging results, the possible beneficial effect of G-90 extracted from earthworm on peripheral nerve regeneration still remains unclear and requires further investigation and confirmation.

## 9. Neural Cell Adhesion Molecule

The neural cell adhesion molecule (NCAM) is a member of the immunoglobulin superfamily. Several studies showed that NCAM-induced neurite outgrowth depends on Ras-MAPK pathway activation [77]. NCAM-dependent cell migration to fibronectin required an intact MEK-ERK signaling pathway [78]. The putative adhesins of the immunoglobulin superfamily presumably from dilong extract could promote migration by MEK-ERK pathway activation. These bioactive compounds may indirectly cause ERK activation or directly activate plasminogen to plasmin by fibrinolytic enzyme, resulting in Schwann cell migration promotion during nerve regeneration.

## 10. Action of Dilong Extract

We suggest that dilong extract promotes proliferation by allowing Schwann cell survival. The IGF-IGFIR-Akt-Bcl2 axis stimulates tissue growth [21] and axonal regeneration [26]. IGF in certain cells, such as hematopoietic cells, functions as an inhibitor of cell death [79]. Activation of the PI3K/Akt pathway promotes cell survival. Activation of Akt leads to the phosphorylation of Bad [80] and connects a proximal survival signal with the Bcl-2 family to protect against apoptosis. Results of the immunoblotting assay showed that PI3K siRNA blocked the earthworm extract-induced expression of the anti-apoptotic proteins pBad and Bcl2. Cell cycle profiles were obtained for earthworm extract-treated Schwann cells after transfection with PI3K siRNA and for treated cells that were not transfected with PI3K siRNA. Knockdown of PI3K led to a significant inhibition of DNA synthesis in cells treated with earthworm extract for 24 h as evidenced by the fact that the number of S phase proliferating cells decreased. These data indicate that Schwann cell survival and proliferation are PI3K-dependent processes that are mediated, atleast in part, by IGF-I.

## 11. The Future of Nerve Cell Regeneration and Possible Influence of Dilong

The findings mentioned previously provide researchers another novel function during neuron regeneration. Although the treatment benefits of earthworms have been strongly supported, more precise studies are still required to: (i) reveal the precise and complete biochemical profile and (ii) suggest accurate doses of medication. In Boyd's study, the results suggested a dose-dependent facilitation and inhibition of peripheral nerve regeneration by brain-derived neurotrophic factor (BDNF). In contrast to the low-dose group, the high doses of BDNF (12–20 *μ*g day^−1^ for 28 days) significantly inhibited motor axonal regeneration [81]. Chang's study suggested similar results [51]. As a word of caution, an excessive earthworm extract load in the medium could provoke an adverse response to recovery of neuron regeneration. In other words, there are threshold dosages above and below a certain effective dose. The findings of our study provide another novel function during neuron regeneration. However, the nerve growth-suppressing action by high doses of earthworm extract at concentrations of 250–1000 mg mL^−1^, indicates that an excessive earthworm extract load in the medium could provoke an adverse response to neuron regeneration recovery. It demonstrates that excessive supplement could saturate the neurotrophin receptor, P75, to block the neuron regrowth-promoting function. Therefore, an appropriate dose of earthworm extract should be carefully selected to reach the highest potential for enhanced Schwann cell migration.

Based on these findings, we believe that certain components of earthworm extract can exert cell migration, proliferation and survival activity. The results demonstrate that dilong extract can stimulate Schwann cell migration and upregulate PAs and MMP2/9 expression mediated through the MAPK pathways, ERK1/2 and p38. Earthworm extract also stimulates Schwann cell proliferation and survival through the PI3K/Akt system mediated by IGF-I. The activity of dilong extract is probably related to its ability to induce G_1_ phase cell cycle progression by altering the expression of proteins that control the cell cycle (cyclin D1, cyclin E and cyclin A), resulting in the upregulation of the anti-apoptotic Bcl_2_ protein. Further analyses are essential to determine the presence of other bioactive compounds in dilong extract that might promote cell migration, survival and proliferation as well as the optimal dose of dilong extract.

## Funding

This work was supported by grants from the China Medical University Hospital (DMR-96-047), 1PT Biotechnology Co., Ltd., and the China Medical University (CMU95-058, CMU95-060, CMU96-102 and CMU97-CMC-007) and supported in part by Taiwan Department of Health Clinical Trial and Research Center of Excellence (DOH99-TD-B-111-004).
